# Postoperative Complications of Upfront Ovarian Cancer Surgery and Their Effects on Chemotherapy Delay

**DOI:** 10.3390/curroncol31090417

**Published:** 2024-09-19

**Authors:** Julia Heikkinen, Henna Kärkkäinen, Marja-Liisa Eloranta, Maarit Anttila

**Affiliations:** 1 Department of Obstetrics and Gynecology, Kuopio University Hospital, 70210 Kuopio, Finland; henna.karkkainen@pshyvinvointialue.fi (H.K.); maarit.anttila@pshyvinvointialue.fi (M.A.); 2 Department of Obstetrics and Gynecology, Central Finland’s Hospital Nova, 40620 Jyväskylä, Finland; marja-liisa.eloranta@hyvaks.fi

**Keywords:** advanced ovarian cancer, primary debulking surgery, postoperative complications

## Abstract

**Simple Summary:**

Upfront surgery to resect all visible tumors, followed by adjuvant chemotherapy, is the cornerstone of treating advanced ovarian cancer. In this retrospective study, 172 patients underwent primary debulking surgery; we analyzed the postoperative complications and complication rates and examined the effects of complications on adjuvant treatment. This study shows that complications are common after extensive surgery; however, most complications can be treated effectively, and delay in adjuvant treatment is rare.

**Abstract:**

Background: Extensive surgery on advanced-stage epithelial ovarian cancer is associated with increased postoperative morbidity, which may cause a delay in or omission of chemotherapy. We examined postoperative complications and their effects on adjuvant treatment in patients undergoing primary debulking surgery (PDS). Methods: Stage IIIC-IV epithelial ovarian cancer patients who underwent PDS between January 2013 and December 2020 were included. Patients were divided into two groups according to the radicality of the operation, i.e., extensive or standard surgery, and their outcomes were compared. Results: In total, 172 patients were included; 119 underwent extensive surgery, and 53 had standard surgery. Clavien–Dindo grade 3–5 (CDC 3+) complications were detected in 41.2% of patients after extensive operations and in 17% after standard surgery (*p* = 0.002). The most common CDC 3+ complication was pleural effusion. Despite the difference in the complication rates, the delay in chemotherapy did not differ between the extensive and standard groups (*p* = 0.98). Conclusions: Complications are common after PDS. Extensive surgery increases the complication rate, but most complications can be treated effectively; therefore, a delay in adjuvant treatment is rare.

## 1. Introduction

Ovarian cancer is the sixth most common cancer in women and the leading cause of death by gynecological malignancies [1] Since it lacks early symptoms or a predictive screening method, ovarian cancer is usually diagnosed in an advanced stage, when the disease has spread to the retroperitoneal lymph nodes and/or above the pelvic brim, to the hepatic and/or splenic parenchyma, or to extra-abdominal organs, which the International Federation of Gynecology and Obstetrics (FIGO) classifies as stages III–IV [2].

The treatment for advanced ovarian cancer is based on either primary (PDS) or interval (IDS) cytoreductive surgery, combined with postoperative taxane- and platinum-based chemotherapy. According to guidelines, PDS is considered the treatment of choice if good surgical outcomes can be achieved, but IDS is typically less surgically demanding and less prone to complications; it may be more beneficial in non-fit patients, or if the tumor burden is high and has spread to sites where it cannot be resected (stage IV disease) [3,4]. The amount of residual tumor is an important prognostic factor for survival [5,6]. According to the Gynecological Oncology Group, the surgical outcome is classified as complete if no visible residual tumor is left. If the largest diameter of the residual tumor is between 1 and 10 mm, the surgical outcome is classified as optimal, and operations leaving a residual tumor larger than 10 mm are suboptimal.

Conventionally, the standard cytoreductive surgery for ovarian cancer has included bilateral salpingo-oophorectomy and hysterectomy; gastrocolic omentectomy; removal of enlarged lymph nodes; and, if needed, en bloc resection of the rectosigmoid bowel. Complete cytoreduction is a remarkable independent prognostic factor for overall survival [5,6,7]. In a review of 13 papers, achieving a complete surgical outcome in stage III-IV disease improved the median overall survival up to 70 months in patients with optimal, and 30 months in those with suboptimal, operative outcomes [8]. Since most patients have the disease spread above the pelvic brim, this goal is usually unachievable without upper abdominal procedures. This has led to the implementation of extensive surgery as a crucial part of ovarian cancer treatment. Extensive surgery includes at least one of the following procedures in addition to the standard operation: diaphragmatic peritonectomy or partial diaphragmatic resection, extensive peritonectomy, splenectomy, cholecystectomy, pancreatic resection, multiple bowel resections, gastrectomy, or liver resection.

Despite the crucial role of cytoreductive surgery, adjuvant chemotherapy is also mandatory in treating advanced ovarian cancer. Extensive surgery increases the complication rate, and the number of additional procedures correlates with morbidity [9,10]. Severe complications and a longer recovery time can cause a delay in or even an omission of chemotherapy [11].

Even though extensive surgery has been widely accepted in cytoreductive surgery of ovarian cancer, data outside of pioneering centers are still limited. Most complication-related data are based on interventional trials and studies conducted in highly specialized units [6,7,8,9,10]. Patient characteristics and clinical resources in these centers may differ from those for unselected real-world patient cohorts. This study aims to evaluate postoperative complications and their impacts on starting adjuvant chemotherapy in women undergoing primary debulking surgery in one tertiary referral center with an intermediate number of primary ovarian cancer surgeries per year, according to the European Society of Gynaecological Oncology guidelines [12].

## 2. Methods

**Figure 1:**
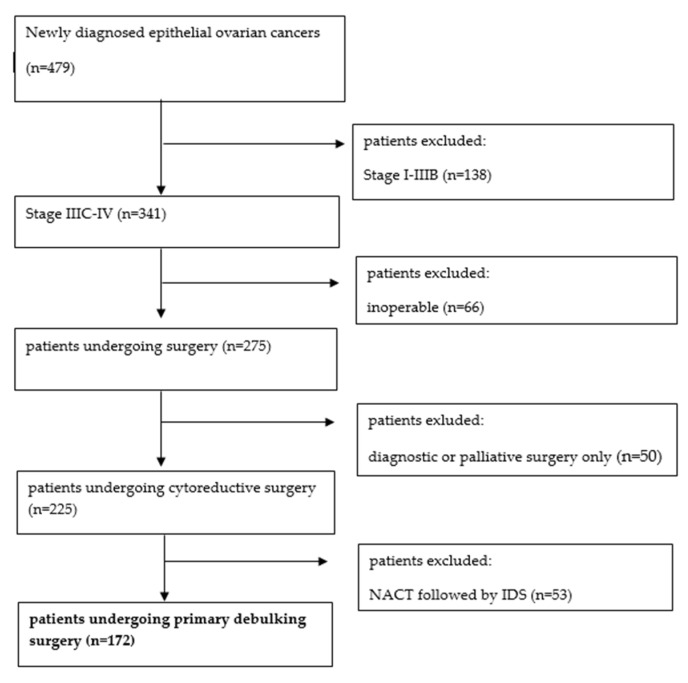
Patient inclusion and exclusion flow chart.

Data were collected retrospectively from patients newly diagnosed with advanced epithelial ovarian, fallopian tube, or primary peritoneal cancer between January 2013 and December 2020 at Kuopio University Hospital, a tertiary referral center in Eastern Finland. Only patients diagnosed with stage IIIC or IV disease, according to the International FIGO classification (2014), were included [13]. The histological diagnosis was made by a pathologist dedicated to gynecological pathology. Inoperable patients and patients with non-epithelial ovarian tumors or borderline tumors, as well as those undergoing neoadjuvant chemotherapy or only diagnostic or palliative surgery, were excluded from our study. The inclusion and exclusion criteria are presented in the flow chart below (Figure 1). This study was conducted according to the standards of the local ethics committee of Kuopio University Hospital, Finland (Nr. 376/2016).

The patients underwent transvaginal ultrasound examination performed by a gynecologist or gynecological oncologist, and, if malignancy was suspected, further imaging was conducted. The preoperative evaluation was performed by a radiologist dedicated to gynecological oncology with several years of experience. According to guidelines, all patients underwent computed tomography (CT) of the chest, abdomen, and pelvis, and most of them also had magnetic resonance imaging (MRI) of the pelvis/abdomen to evaluate the disease burden of the bowel and to produce data for another research aim [14]. The data collected preoperatively included each patient’s age, body mass index (BMI), American Society of Anesthesiologists (ASA) preoperative score, and preoperative levels of serum CA125.

Treatment was discussed in a multidisciplinary meeting. Based on their characteristics, performance status, and the extent of their disease, patients were selected for either primary debulking surgery (PDS) or neoadjuvant chemotherapy (NACT) and interval debulking surgery (IDS). Generally, PDS was selected if a patient had a sufficient performance state and complete resection was anticipated according to the preoperative radiologic evaluation. However, if complete resection could not be achieved during operation, optimal resection was also considered acceptably operable, based on performance and whether an adequate cytoreduction was achievable based on studies showing positive effects after optimal resection compared to suboptimally resected patients [5,6].

Patients usually underwent a straight midline laparotomy, but, if resectability was uncertain, a diagnostic laparoscopy was made. Diagnostic laparoscopies were converted into laparotomies if the gynecological oncologist presumed that the surgical goals could be achieved; otherwise, they remained diagnostic laparoscopies, and the patient was excluded from our study. Cytoreductive surgery was performed or supervised by an experienced gynecological oncologist, and upper abdominal parenchymal resections and bowel operations were performed in cooperation with a colorectal surgeon.

The operation was considered extensive when at least one of the following procedures was performed: diaphragmatic peritonectomy or resection, large peritonectomy, splenectomy, cholecystectomy, pancreatic resection, gastrectomy, liver resection, or multiple bowel resections. In a standard operation, bilateral salpingo-oophorectomy, hysterectomy, and, if needed, en bloc resection of the rectosigmoid bowel were performed with or without pelvic and/or para-aortal lymphadenectomies. Surgical complexity was assessed with the surgical complexity score (SCS) [15]. Postoperative complications were recorded according to the Clavien–Dindo classification.

Patient demographic data, laboratory values (CA125, serum albumin), surgical procedures performed, operation duration, blood loss, histological data, postoperative complications, and length of hospital stay were retrieved from institutional medical records.

Categorical variables were compared with a chi-square test. Parametric and non-parametric continuous variables were compared with a two-sample impaired *t*-test and a Mann–Whitney U test, respectively. Factors predicting postoperative complications were evaluated using multivariate models. Logistic regression was performed for variables with a *p*-value < 0.1 based on the chi-square test. A *p*-value < 0.05 was considered statistically significant. SPSS version 26 was used for all statistical analyses.

## 3. Results

During the study period, 479 new epithelial ovarian cancer patients were diagnosed. A total of 172 patients met the inclusion criteria and were considered for the analysis.

Of the included patients, 119 (69.2%) underwent extensive surgery, and 53 (30.8%) had standard surgery.

The baseline characteristics of the patients are shown in Table 1. The proportion of patients aged ≥70 years undergoing extensive surgery was 27.1%, compared with 46.2% undergoing only standard surgery (*p* = 0.009). In the extensive surgery group, an ASA score ≤2 was seen in 68.1%, compared with 41.5% in the standard group (*p* = 0.002), and the SCSs were ≥8 in 63.0% and 0%, respectively (*p* < 0.001). There were no statistical differences in other tested parameters.

After extensive surgery, the complete, optimal, and suboptimal resection rates were 54.2%, 37.3%, and 8.5%, respectively, and after standard surgery, the resection rates were 49.1%, 11.3%, and 39.6%, respectively. The differences in resection rates were statistically significant (*p* < 0.001).

In total, 285 extensive procedures were performed on 119 patients. The most common extensive procedure was diaphragmatic peritonectomy, which was performed on 57.0% (*n* = 98) of the women. Thirty-seven (37.8%) of these women had either pleural resection or an unintentional opening of the pleural cavity due to diaphragmatic peritonectomy. The second most common procedure was extensive peritonectomy, which was performed on 49.4% (*n* = 85) of the women. All procedures are listed in Table 2. There was one intraoperative death, due to a laceration of the vena cava during a diaphragmatic peritonectomy.

The mean operation time was 366 min; the mean estimated blood loss was 1345 mL; and the mean length of hospitalization was 8.4 days. However, significant differences were found in these parameters between the extensive and standard groups, as the mean operation time was 403.1 min in the extensive group, compared with 284.1 min in the standard group (*p* < 0.001). The mean blood loss rates were 1543.2 mL vs. 901.4 mL, respectively (*p* < 0.001), and the mean lengths of hospitalization were 9.02 days vs. 7.08 days, respectively (*p* < 0.001). There was no difference in the reoperation rate between the extensive and standard groups (Table 3).

**Figure 2:**
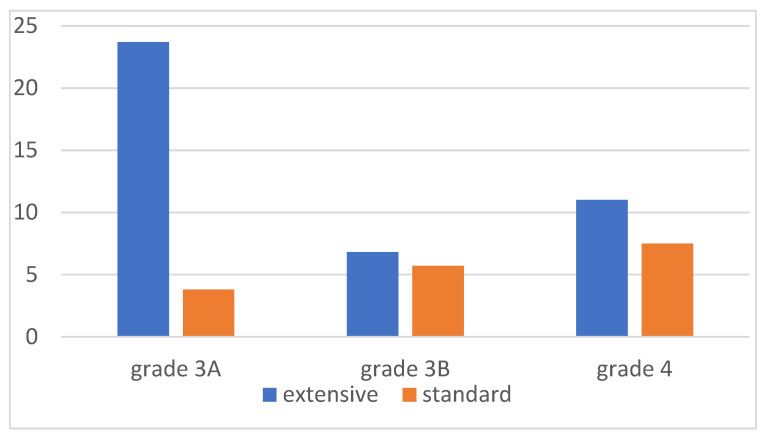
Clavien–Dindo grade 3+ complications (%).

Severe complications (Clavien–Dindo classification (CDC) grades 3–4) were detected in 58 (33.9%) patients within 30 days of surgery. Of these, 52.6% were grade 3A complications, mainly pleural effusions (Figure 2). After extensive surgery, 49 (41.2%) patients were reported to have CDC 3–4 complications, compared with 9 (17.0%) patients with standard operations (*p* = 0.002). There were no grade 5 postoperative complications. The most common severe complication was pleural effusion (18.0%). Pleural effusion was detected in 28.3% of the patients undergoing only diaphragmatic peritonectomy and in 35.1% on whom diaphragmatic resection was performed. CDC 3+ complications and their frequencies are listed in Table 4. Additionally, there were 17 deep venous or pulmonary thromboses, and one suspected anastomotic leakage was treated without reoperation. The overall need for Intensive Care Unit (ICU) admission was 9.4%. The difference in ICU admission rates was not statistically significant between extensive and standard operations.

We analyzed the predictive parameters for severe complications after extensive surgery. Only SCS (*p* = 0.002), estimated blood loss (*p* = 0.011), and the length of the operation (*p* < 0.001) predicted severe complications in univariate analysis. The procedures associated with severe complications were diaphragmatic peritonectomy (*p* = 0.002), rectosigmoid resection (*p* = 0.004), and multiple bowel resections (*p* = 0.03) (Table 5). When these variables were analyzed in multivariate analysis, operative time (*p* = 0.004) was the only factor associated with severe complications. Every 10 min increase in operation time was associated with a 6% increase in complication rate (Table 6). As expected, women who suffered from severe complications had significantly longer hospital stays than women with mild to no complications: 12.0 days vs. 6.6 days, respectively (*p* < 0.001). The median time between the operation and the start of chemotherapy was 22 days when a woman had mild to no complications, and 28 days if there were severe complications. Of the patients, 47 (29.0%) had chemotherapy postponed by more than 28 days, and 7 (4.3%) had it postponed by more than 42 days. Postponing chemotherapy was significantly more common among women with severe complications (*p* = 0.003 for postponing >28 days, and *p* = 0.029 for >42 days, respectively). Anastomotic leakage had a significant impact on chemotherapy postponement (*p* < 0.001 and *p* = 0.002 for >28 and >42 days, respectively). Extensive surgery was not associated with chemotherapy postponement (*p* = 0.16 for >28 days, and *p* = 0.98 for >42 days). Six women never recovered enough for chemotherapy (Supplement Table S1). Four of these women had severe complications, and two had undergone extensive operations.

## 4. Discussion

Complete cytoreduction is one of the most important prognostic factors in managing ovarian, tubal, and primary peritoneal cancers [5,6,16]. This has led to the implementation of more aggressive surgeries during the past decade [17,18]. This study shows that upper abdominal procedures are common when aiming for complete surgical results, and resection rates were significantly higher in women who had extensive surgery. Nonetheless, certain factors could have influenced the results. If the tumor burden is in a wider range than expected, unresectable because of anatomical location, or requires extensive resections that could lead to a high risk of complications, the surgeon may choose to perform only standard surgery. The patients in the standard group were older and had higher ASA scores. These are known risk factors for impaired postoperative recovery and must be considered when offering personalized treatment [19]. It is critical to keep in mind the limitations of preoperative imaging [20].

In our study, the complete resection rate was 52.7%, and the optimal resection rate was 29.6% after PDS. In previous studies, the complete resection rate after PDS was 45.5–75.1%, and the optimal resection rate after PDS was 20.7–39% [21,22]; thus, our resection rates are within the ranges of those from previous results.

Even though a wide selection of upper abdominal procedures may be performed in modern ovarian cancer surgery, the most frequent procedures are diaphragmatic and extensive peritonectomies [22,23,24]. A similar finding was also seen in our study, as 57.0% of the patients underwent diaphragmatic peritonectomies, and 49.4% underwent extensive peritonectomies.

More aggressive surgery increases the risk of complications. In our study, the severe complication rate was 28.8%. Our study showed the effects of surgical complexity and more demanding surgery, as 41.9% of the women who underwent extensive surgery had CDC 3–4 complications, compared with 15.9% in women who had only standard surgery. Postoperative mortality was 0%. The complication rate significantly varies in the literature. In studies of advanced-stage epithelial ovarian cancer (EOC) patients undergoing PDS, morbidity during the first 30 days is 9.51–22.3%, and postoperative mortality is 0.8–4.6% [5,21,25,26,27].

The number of patients with PDS is likely a major factor in explaining the difference in complication rates. At our hospital, 75.2% underwent PDS, compared with 30.9–69.5% in previous studies [26,27]. Notably, most papers studying PDS do not represent the percentages of patients who undergo PDS. As the role of NACT is still under debate, we have favored PDS at our hospital. Two well-known RCTs (EORTC-GCG and CHORUS) showed no difference in overall survival between PDS and IDS, but fewer complications in the IDS group [28,29]. However, these RCTs have been criticized for low complete cytoreduction rates in both study arms. The SCORPION trial was designed to overcome certain weaknfagottiesses in earlier RCTs, and the complication rate was significantly lower in its NACT/IDS group. Overall survival (OS) and progression-free survival were slightly better in the PDS group, but the difference was not statistically significant [30]. On the other hand, in retrospective studies, PDS has been associated with significantly improved OS [26,31]. A meta-analysis including 16 studies and 57,450 patients showed increased OS after PDS compared with NACT, despite the increased completeness of debulking, and the reduced risk of postsurgical death and major infections, in the NACT group [32]. The ongoing TRUST Trial will likely increase our knowledge of the preferred approach [33].

Laparotomy is the gold standard in primary debulking surgery for advanced ovarian cancer (stage III–IV), even though there are some small retrospective series of patients undergoing minimally invasive surgery (MIS) in a PDS setting [34,35]. One of these studies reported a complication rate of 18%. Also, overall survival compared to laparotomy is still unknown. In the treatment of ovarian cancer, MIS is commonly used for diagnostic purposes to identify inoperable tumor dissemination. MIS can be considered in the treatment of early ovarian cancer, and in selected cases after NACT or in tertiary surgery [36].

Even though complications are relatively common after extensive surgery, many are easily treatable. Almost one-quarter of the complications were pleural effusions, leading to pleural drainage. This complication is specifically related to extensive surgery and diaphragmatic peritonectomy. In the literature, pleural effusion has been detected in 23–27% of patients after upper abdominal surgery [22,23]. Diaphragmatic peritonectomy or resection and liver mobilization are known risk factors for pleural effusion, and the relevance of placing a chest tube intraoperatively has been discussed [37]. Our data show that the incidence of pleural effusion after pleural resection was 35.1%. Therefore, intraoperative pleural drainage seems unjustified, as most patients would not benefit from the chest tube.

Since the effect of complete cytoreduction on OS is remarkable, the limits of extensive surgery have moved forward, and the feasibility and importance of extra-abdominal surgery are being investigated. Paraphrenic and hepatoceliac lymph node resections seem feasible with acceptable morbidity in selected patients [38,39]. Nevertheless, the evidence for survival benefits is still lacking, as the few small-sample studies are conflicting [40,41].

It is important to identify patients who benefit the most from this surgery, without compromising the surgical results because of unbearable complications. Earlier studies have found several factors that affect postoperative complication rates. These include age, smoking, serous histology, FIGO stage, ASA score, WHO performance status, the presence of ascites, preoperative albumin, operative time, and surgical complexity [21,23,25]. In our study, none of the tested preoperative parameters were associated with increased postoperative complications because of a small sample size or preoperative patient selection between PDS and IDS. Extensive surgery, high SCS, operative time, and estimated blood loss correlated with postoperative complications, but, after multivariate analysis, only operative time remained statistically significant.

Even though the residual tumor amount is the most significant factor for OS in advanced EOC, chemotherapy remains essential in treatment. Some papers have evaluated the optimal interval between the onset of chemotherapy and surgery. In a study by Paulsen et al., initiating chemotherapy within 4 weeks after surgery slightly improved OS, but the difference was not statistically significant [42]. In studies by Wright et al. and Seagle et al., the delay in chemotherapy was >6 weeks, and in a study by Tewari, delays beyond 36 days seemed to decrease OS [43,44,45]. Singh et al. studied preoperative risk factors in postponing chemotherapy. An age >65 years, albumin level <3.5, and comorbidities were associated with a delay [46]. All are risk factors for postoperative complications, indicating the vulnerability of this patient group.

In our study, even though postponing chemotherapy was significantly more common in women with postoperative complications, the type of surgery, i.e., extensive or standard, was not associated with a delay in starting chemotherapy. However, contradictory findings have been shown in the literature; for example, Benedetti Panici et al. found that initiating chemotherapy did not differ between complicated and uncomplicated surgeries [22].

In a study by Grimm et al., the anastomotic leakage rate was higher among patients undergoing rectosigmoid resection than patients undergoing other bowel resections [47]. Notably, anastomotic leakage was the only complication in our study that led to statistically significant adjuvant treatment postponement.

## 5. Conclusions

Our study presents real-life data on unselected patients, showing that PDS is feasible among many patients. Extensive procedures are often needed in EOC surgery, and more aggressive surgery increases the risk of postoperative complications. Owing to the peritoneal spread of this disease, diaphragmatic peritonectomy and extensive peritonectomy of the abdominal cavity are the most common extensive procedures, with pleural effusion as the most common complication after extensive surgery. Most postoperative complications can be treated effectively, without delays in adjuvant treatment. Nonetheless, anastomotic leakage is related to more clinically significant consequences, including the risk of postponing chemotherapy.

## Figures and Tables

**Table 1:** Patient characteristics and tumor histology.

Characteristic	Extensive Group	Standard Group	*p*-Value
Age ≥ 70 years	32 (27.1%)	24 (46.2%)	0.009
BMI ≥ 35 kg/m^2^	7 (6.1%)	3 (5.9%)	0.692
CA12-5 > 1000 kU/L	33 (28.2%)	8 (15.4%)	0.054
Serum albumin < 30	26 (24.1%)	8 (20.5%)	0.543
ASA score			0.002
1	19 (16.0%)	4 (7.5%)	
2	62 (52.1%)	18 (34.0%)	
3	36 (30.3%)	30 (56.6%)	
4	2 (1.7%)	1 (1.9%)	
FIGO stage			0.055
IIIC	58 (48.7%)	34 (64.2%)	
IVA	7 (5.9%)	3 (5.7%)	
IVB	54 (45.4%)	16 (30.2%)	
Histology			0.100
High-grade serous	99 (83.2%)	36 (67.9%)	
Low-grade serous	7 (5.9%)	2 (3.8%)	
Mucinous	1 (0.8%)	1 (1.9%)	
Endometrioid	4 (3.4%)	3 (5.7%)	
Clear cell	2 (1.7%)	3 (5.7%)	
Other	6 (5.0%)	6 (11.3%)	
Mixed	0	2 (3.8%)	
SCS			<0.001
Low ≤ 3	3 (2.5%)	22 (42.3%)	
Intermediate 4–7	41 (34.5%)	30 (57.7%)	
High ≥ 8	75 (63.0%)	0	
BRCA positive	16.3%	11.1%	0.165

**Table 2:** Numbers and percentages of the performed procedures.

Procedure	*n* (%)
Diaphragmatic peritonectomy	98 (57.0%)
Extensive peritonectomy	85 (49.4%)
Rectosigmoid resection	74 (43.0%)
Splenectomy	39 (22.7%)
Diaphragmatic resection	37 (21.5%)
Minor liver operation	24 (14.0%)
Multiple bowel resections	13 (7.6%)
Cholecystectomy	12 (7.0%)
Liver resection	10 (5.8%)
Pancreatic resection	4 (2.3%)
Gastrectomy	0 (0%)

**Table 3:** Operative results.

	Extensive Group	Standard Group	*p*-Value
Residual disease			
Complete (R = 0)	53.8%	49.1%	<0.001
Optimal (R < 10 mm)	37.0%	11.3%	
Suboptimal (R ≥ 10 mm)	8.4%	39.6%	
Mean operation time (min)	403.1	284.1	<0.001
Mean estimated blood loss (mL)	1543.2	901.0	<0.001
Mean hospital stay (days)	9.02	7.08	<0.001
Clavien–Dindo 3–4 complication (*n*/%)	49/41.2%	9/17.0%	0.002
ICU admission	11.0%	5.7%	0.266
Reoperation	9.3%	9.4%	0.981

**Table 4:** Postoperative complications in the first 30 days after surgery.

Complication	N	%
Pleural effusion	27	16.0
Anastomotic leakage	9	5.3
Wound opening	4	2.4
Intra-abdominal abscess	4	2.4
Septicaemia	3	1.8
Ileus	3	1.8
Seroma	3	1.8
Atrial fibrillation	3	1.8
Ascites	3	1.8
Pneumonia	2	1.2
Wound infection	2	1.2
Postoperative infection	2	1.2
Rectovaginal fistula	2	1.2
Pneumothorax	1	0.6
Fascial dehiscence	1	0.6
Pancreatic leakage	1	0.6
Haematoma (intra-abdominal)	1	0.6
Acute renal insufficiency	1	0.6
Peritonitis	1	0.6
Urinary tract injury	1	0.6
Biliary leakage	1	0.6
Angina pectoris	1	0.6
Acute myocardial infarction	1	0.6
Pulmonary oedema	1	0.6
Thrombosis of a. brachialis	1	0.6
Myasthenic crisis	1	0.6
ARDS	1	0.6

**Table 5:** Factors related to Clavien–Dindo 3+ complications.

	*p*-Value
Age ≥ 70	0.34
BMI > 35	0.75
CA125 > 1000	0.13
Albumin < 30	0.54
Serous histology	0.16
ASA score 3–4	0.76
Extensive surgery	0.002
Diaphragmatic peritonectomy	0.003
Extensive peritonectomy	0.42
Rectosigmoid resection	0.002
Splenectomy	0.15
Diaphragmatic resection	0.57
Minor liver operation	0.33
Cholecystectomy	0.16
Multiple bowel resections	0.034
Pancreatic resection	0.50
Liver resection	0.33
Estimated blood loss	<0.001
Operation time	<0.001
SCS	0.002

**Table 6:** Multivariate analysis of factors associated with postoperative complications.

	OR (95% CI)	*p*-Value
Extensive surgery	1.71 (0.47–6.27)	0.42
Diaphragmatic resection	0.95 (0.30–2.95)	0.92
Rectosigmoid resection	1.19 (0.48–2.93)	0.71
Estimated blood loss	1.00 (1.00–1.01)	0.19
Operation time	1.006 (1.002–1.010)	0.004
SCS	0.97 (0.40–2.34)	0.94

## Data Availability

The data presented in this study are available on request from the corresponding author, due to privacy and legal restrictions under the Finnish Act on the Secondary Use of Health and Social Data, which imposes specific conditions on the transfer and processing of data.

## Supplementary Materials

The following supporting information can be downloaded at: https://www.mdpi.com/article/10.3390/curroncol31090417/s1, Table S1: Data from the patients who did not receive adjuvant chemotherapy.
