# Supplementary material for: Postoperative Complications of Upfront Ovarian Cancer Surgery and Their Effects on Chemotherapy Delay
Source: Curr Oncol. 2024 Sep 19;31(9):5630–42. doi: 10.3390/curroncol31090417 (PMC11430809; doi:10.3390/curroncol31090417)
Supplement: Supplementary file 1 [file curroncol-31-00417-s001.zip › curroncol-3092386-supplementary.pdf]

Supplement 1

Data from patients who did not receive adjuvant chemotherapy.

	Age (years)	Type of surgery	Complication
Patient 1	62	extensive	postoperative ileus, septicemia, rapid progression
Patient 2	73	standard	Alzheimer's disease, poor general condition
Patient 3	69	standard	anastomotic leakage, difficult postoperative infection, disease progression
Patient 4	80	standard	poor general condition
Patient 5	59	standard	poor general condition
Patient 6	71	extensive	anastomotic leakage, difficult postoperative infection
